# A Naturally Occurring Polymorphism at *Drosophila melanogaster Lim3* Locus, a Homolog of Human *LHX3/4*, Affects *Lim3* Transcription and Fly Lifespan

**DOI:** 10.1371/journal.pone.0012621

**Published:** 2010-09-07

**Authors:** Olga Yu. Rybina, Elena G. Pasyukova

**Affiliations:** Institute of Molecular Genetics of RAS, Moscow, Russia; National Institute on Aging, United States of America

## Abstract

*Lim3* encodes an RNA polymerase II transcription factor with a key role in neuron specification. It was also identified as a candidate gene that affects lifespan. These pleiotropic effects indicate the fundamental significance of the potential interplay between neural development and lifespan control. The goal of this study was to analyze the causal relationships between *Lim3* structural variations, and gene expression and lifespan changes, and to provide insights into regulatory pathways controlling lifespan. Fifty substitution lines containing second chromosomes from a *Drosophila* natural population were used to analyze the association between lifespan and sequence variation in the 5′-regulatory region, and first exon and intron of *Lim3A*, in which we discovered multiple transcription start sites (TSS). The core and proximal promoter organization for *Lim3A* and a previously unknown mRNA named *Lim3C* were described. A haplotype of two markers in the *Lim3A* regulatory region was significantly associated with variation in lifespan. We propose that polymorphisms in the regulatory region affect gene transcription, and consequently lifespan. Indeed, five polymorphic markers located within 380 to 680 bp of the *Lim3A* major TSS, including two markers associated with lifespan variation, were significantly associated with the level of *Lim3A* transcript, as evaluated by real time RT-PCR in embryos, adult heads, and testes. A naturally occurring polymorphism caused a six-fold change in gene transcription and a 25% change in lifespan. Markers associated with long lifespan and intermediate *Lim3A* transcription were present in the population at high frequencies. We hypothesize that polymorphic markers associated with *Lim3A* expression are located within the binding sites for proteins that regulate gene function, and provide general rather than tissue-specific regulation of transcription, and that intermediate levels of *Lim3A* expression confer a selective advantage and longer lifespan.

## Introduction

Lifespan is determined by a complex interplay between environmental and genetic factors. Temperature, air pollution, nutrition, and other factors affect multiple processes through various signaling and metabolic pathways. Many genes are involved in these pathways, and therefore control lifespan. Indeed, hundreds of genes are known to affect lifespan in model organisms [1]–[3]. However, many aspects of the genetic control of lifespan remain unclear. One that is especially interesting for us is how naturally occurring structural and functional variations in a gene can affect this phenotypic trait. Recent studies of natural nucleotide divergence in a variety of *Drosophila* genes demonstrated associations between structural polymorphisms in several genes and quantitative traits, including lifespan [4]–[6]. However, the causal relation of these structural variations and gene expression changes and phenotype alterations remains poorly understood.

Several candidate genes affecting lifespan have been revealed using recombination mapping followed by quantitative complementation tests with deficiencies and mutations at candidate loci [7]. Among others, *Lim3* was identified as a candidate gene affecting lifespan [8]. Recent data show that this gene is also associated with locomotion behavior [9].

*Lim3* is located in cytological region 37B13-37C1 of the second chromosome, and is a homeobox gene that encodes an RNA polymerase II transcription factor (TF) required for development and function of neurons. *Lim3* is involved in complicated motor neuron specification networks, and is activated by *Nkx6* and repressed by *Even skipped* (*Eve*) [10]. *Lim3* may regulate axon extension and fasciculation through its downstream target, *FasciclinIII* [11]. With Islet and Drifter, Lim3 constitutes a “combinatorial code” that generates distinct motor neuron identities [10], [12]. The Lim3 protein contains two LIM domains, a carboxyterminal homeodomain, and a highly conserved 22-amino acid region called the Lim3-specific domain (LSD). Lim3 is highly homologous to the vertebrate LHX3/4 subclass of LIM-homeodomain proteins, with 95% and 98% identity to human LHX3 and LHX4 in the homeodomain region, 89% identity in the LIM domains, and 45% identity in the LSD [13]. Like Lim3, human LHX3/4 are TFs required for pituitary development and motor neuron specification. Mutations in *LHX3/4* are associated with combined pituitary hormone deficiency, rigid cervical spine, or short stature [14]–[16].

The involvement of *Lim3* in both the regulation of neuron development and lifespan control could be of fundamental significance. The effect of Drosophila *Lim3* on lifespan control could be conserved in multicellular eucaryotes, including humans, similar to its role in neuron identification. Analysis of the causal relationships between *Lim3* structure, transcription level, and lifespan will provide insight into conserved regulatory pathways controlling lifespan. In this paper, we demonstrate the potential of naturally occurring polymorphisms in the *Lim3* 5′-regulatory region to modulate gene expression and fly lifespan.

## Results

The exact mechanisms of *Lim3A* transcription, and the structure of its potential regulatory region were unknown. To characterize and evaluate the functional role of naturally occurring polymorphisms of the *Lim3* 5′-regulatory region, we first analyzed initiation of *Lim3A* transcription and determined the exact border between the regulatory and structural parts of the gene, and outlined proximal promoter region and potential binding sites for regulatory proteins within the regulatory region.

### Analysis of Lim3A transcription initiation and proximal promoter region

**Figure 1:**
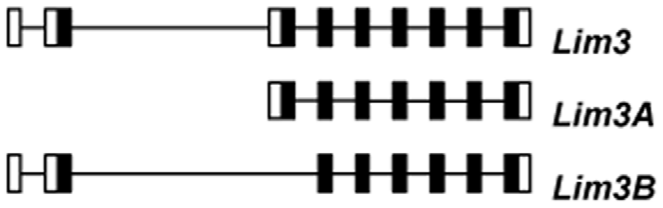
The structure of *Drosophila Lim3* gene. Exons are depicted by rectangles, white rectangles correspond to untranslated regions; introns are indicated by black lines.

*Lim3* was found to produce two mRNAs: *Lim3A* and *Lim3B* (Gen Bank accession nos. NM_057258 and NM_165277), with the same structure, except that the first exon of *Lim3A* is replaced by two different exons in *Lim3B* (Figure 1). We focused on *Lim3A*, which has been shown to have a function in *Drosophila* neuron development [10].

**Figure 2:**
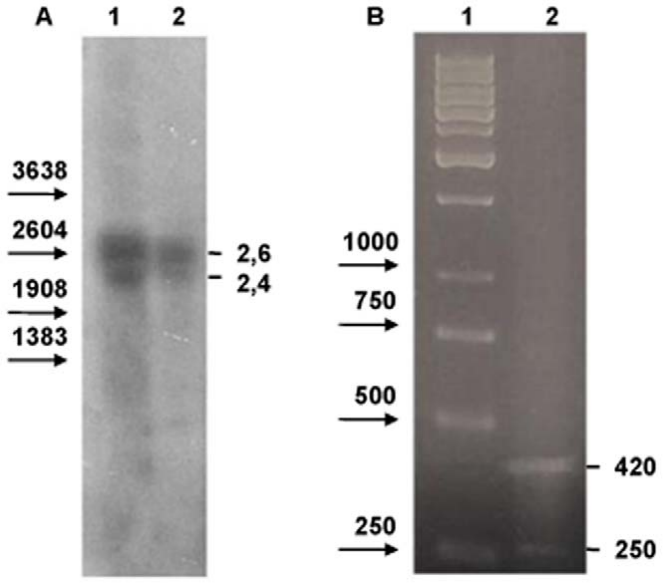
Molecular analyses of *Lim3A*. (A). Northern blot analysis of *Lim3A*. Lanes 1 and 2 present RNA from 12 hr embryos of two different homozygous substitution *Drosophila* lines. PCR fragment including the 5′ region and the first exon of *Lim3A* was used as a probe. The mobilities of standard DNA markers (Promega) are depicted by arrows on the left. The approximate sizes of the transcripts are indicated in kilobases on the right. (B). 5′ RACE analysis of *Lim3A*. Lane 1 is a 1-kb DNA ladder from Fermentas. Lane 2 represents 5′ RACE products, sizes are indicated in base pairs on the right.

**Figure 3:**
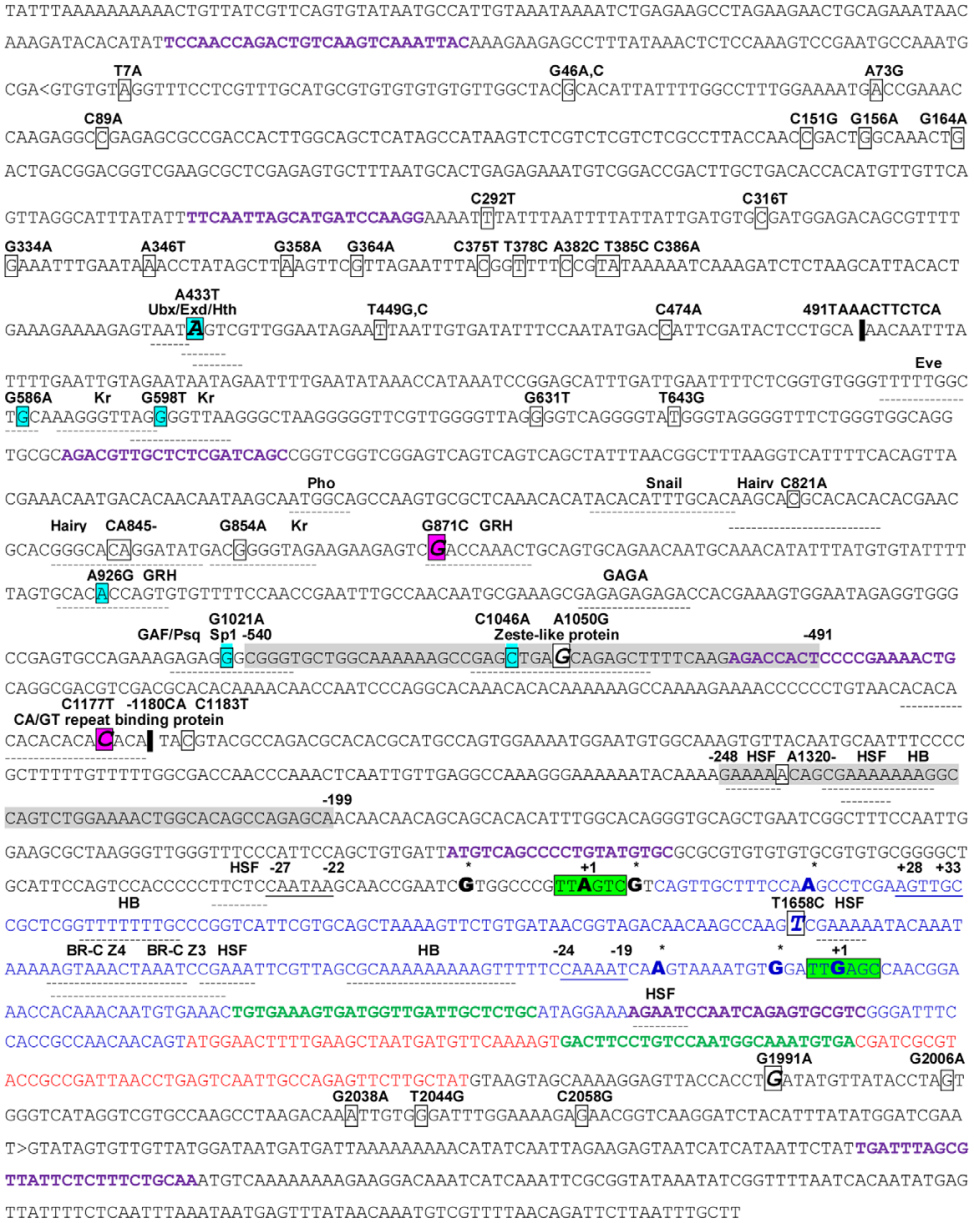
The structure and nucleotide sequence of the 5′ end of *Lim3A* and *Lim3C*. Letters in blue correspond to 5′ UTR of *Lim3A*, letters in red – to translated region of the first exon of *Lim3A*, *Lim3C*. Letters in violet correspond to primers for PCR and sequence analysis. Letters in green correspond to primers for Real-time RT-PCR. The borders of the analysed sequence are depicted by </>. The promoter sequences predicted by the Neural Network Promoter Prediction database, version 2.2 are depicted by light grey color. Sequences marked with green rectangles are initiation regions. TSSs are indicated by large bold letters and asterisks. The underlined sequences are putative core promoter elements. Letters outlined by squares are polymorphic sites which were present in the sample with the frequency 0.06 and higher, insertions are depicted by black rectangles. Large, bold letters in italics outlined by squares are lifespan associated SNPs. Large, bold letters in italics outlined by pink squares are SNPs composing haplotype significantly associated with lifespan. Letters outlined by blue squares are SNPs significantly associated with *Lim3A* mRNA amount. The transcription factor binding sites are denoted by the dotted lines.

Northern blot using a *Lim3A*-specific probe revealed two transcripts (Figure 2A). The larger 2.6 kb major transcript was identical in size to *Lim3A*; the minor 2.4 kb transcript, which we called *Lim3C*, was new. 5′-RACE analysis (Figure 2B) confirmed the additional *Lim3C* mRNA. Sequences of 47 clones obtained by 5′-RACE (GenBank accession no. GU814523–GU814569) demonstrated that each transcript had an array of closely located transcriptional start sites (TSSs) with different initiation rates. The major *Lim3A* TSS (Figure 3) was at −6 nucleotides (18 clones), and the minor TSSs were at −16 (3 clones), −2 (8 clones), and +14 (4 clones) relative to the earlier annotated start site. The major *Lim3C* TSS (Figure 3) was at +184 (8 clones), and the minor TSSs were at +169 (3 clones), and +179 (3 clones) relative to the earlier annotated start site. TSSs located downstream of the major TSS might correspond to accidentally truncated fragments of full-length RNA molecules, so only TSSs represented by three or more clones were considered. *Lim3C* appeared to be 190 bp shorter than *Lim3A* because of the reduced length of the untranslated region (UTR). Seven identical exons were present in both transcripts (data not shown).

*Lim3A* and *Lim3C* TSSs were located within the large 12-kb intron of *Lim3B* (Figure 3). Several bioinformatic resources were used to determine regulatory elements present in the core and proximal promoter regions of *Lim3A* and *Lim3C*. The *Lim3A* transcript start region (initiator) was a close match to the consensus sequence of the *D. melanogaster* initiator T-C-A_+1_-G/T-T-T/C [17], [18], and appeared to be TTA_+1_GTC. Almost identical initiators were found in 13.2% of genes in the *Drosophila* Core Promoter Database. Most of these (63%) contained downstream core promoter elements (DPEs), and mainly had similar functions, specifically RNA polymerase II TF activity, which correlated with the *Lim3* function.

The Eukaryotic Promoter Database, Current Release 100, and *Drosophila* Core Promoter Database were used to detect *Drosophila* core promoter elements in the *Lim3A* regulatory region. A DPE was identified at +28 to +33 nucleotides relative to the major TSS of *Lim3A* (Figure 3). The DPE sequence, AGTTGC, was a reasonable match to the consensus DPE sequence (A/G/T_+28_-C/G-A/T-C/T-A/C/G-C/T) [17], [18], and was encountered in 0.8% of 1926 genes included in the Eukaryotic Promoter Database.

No TATA box was found in the *Lim3A* regulatory region. However, the sequence CAATAA, found at −27 to −22 nucleotides upstream of the *Lim3A* TSS, often occurs at positions from −36 to −21 nucleotides in the regulatory regions of *D. melanogaster* genes (0.6% of 1926 promoter sequences in the Eukaryotic Promoter Database). For example, CAATAA was found in the regulatory regions of five *Enhancer of split* [*E(spl)*] genes (*HLHm3*, *HLHm5*, *HLHm8*, *HLHmβ*, *HLHmγ*) [19], which encode basic helix-loop-helix transcriptional repressors that are expressed mainly during the embryonic stage, and function in neuronal development, similar to *Lim3*. Thus, the CAATAA sequence is common to genes with overlapping expression patterns during embryogenesis [20].

In contrast to *Lim3A*, the *Lim3C* transcription start region TTG_+1_AGC was less similar to the consensus. Promoter elements were not found in the regulatory region of this transcript. The sequence CAAAAT, at −24 to −19 nucleotides relative to the *Lim3C* major TSS, has been found in the regulatory regions of 0.6% of 1926 genes of the Eukaryotic Promoter Database, at a position of −36- to −18 nucleotides, relative to the TSSs.

In addition to the two major and some minor TSSs mentioned above, 5′-RACE analysis revealed TSSs represented by a single clone each, located approximately 250 bp upstream of the *Lim3A* major TSS. These rare long transcripts might use promoters predicted by the Neural Network Promoter Prediction database (Figure 3) at −534 to −485 (score 0.98), and −242 to −193 (score 1.00), relative to the *Lim3A* major TSS or, more likely, are “slippery promoters” typical of both TATA-containing and TATA-less *Drosophila* genes with multiple TSSs [21].

To identify potential TF-binding sites within the proximal regulatory regions of *Lim3A* and *Lim3C*, TFSEARCH version 1.3, MOTIF Search, and other bioinformatic resources (see Materials and Methods) were used. Potential TF binding sites were found for heat-shock factor, which also controls the expression of non-heat shock protein genes, for example, *eve*, in *Drosophila* embryonic development [22]; Hunchback (HB) which is necessary and sufficient for specifying early-born temporal identity in multiple neuroblast lineages [23]; and broad-complex Z3 and broad-complex Z4 (BR-C Z3/ Z4), which are essential for metamorphic reorganization of the central nervous system [24] (Figure 3). HB and BR-C Z3/Z4 are specialized TFs participating in *Drosophila* nervous system morphogenesis that might take part in *Lim3* transcription regulation, which is also essential for neuron development.

### Naturally occurring polymorphisms at Lim3

To determine if *Lim3* function is associated with molecular variation in natural populations of *Drosophila*, we sequenced 2094 bp from 50 alleles from the Raleigh natural population, including 1557 bp of the *Lim3A* regulatory region, 300 bp of the 5′ UTR, 109 bp of the translated region from the first *Lim3A* exon, and 128 bp from the first intron (Table 1, GenBank accession no. 9GU814570, 33GU814571, 40GU814572, 44GU814573, 49GU814574, 58GU814575, 74GU814576, 76GU814577, 77GU814578, 87GU814579, 89GU814580, 98-21GU814581, 98-5GU814582, 100GU814583, 113GU814584, 115GU814585, 122GU814586, 161GU814587, 166GU814588, 180GU814589, 183GU814590, 200GU814591, 201GU814592, 207GU814593, 215GU814594, 226GU814595, 266GU814596, 273GU814597, 284GU814598, 285GU814599, 311GU814600, 316GU814601, 317GU814602, 325GU814603, 327GU814604, 336GU814605, 345GU814606, 351GU814607, 354GU814608, 361GU814609, 369GU814610, 376GU814611, 382GU814612, 407GU814613, 429GU814614, 434GU814615, 444GU814616, 461GU814617, 472GU814618, 473GU814619).

**Table 1:** Parameters of nucleotide diversity in the regulatory region and the beginning of the structural part of the *Lim3A*.

Region	Nucleotide position	Number of Indels +SNPs	π (s. d.)	θ (s. d.)
All sequence	1–2094	16+74	0.00709 (0.00046)	0.00864 (0.00098)
Regulatory region	1–1557	14+58	0.00795 (0.00046)	0.00875 (0.00112)
	1–779	8+39	0.01168 (0.00069)	0.01118 (0.00179)
	780–1557	6+19	0.00421 (0.00043)	0.00631 (0.00135)
Exon	1558–1966	1+7	0.00152 (0.00059)	0.00600 (0.00181)
5′UTR	1558–1857	1+5	0.00181 (0.00078)	0.00670 (0.00223)
Translated region	1858–1966	0+2	0.00073 (0.00070)	0.00410 (0.00290)
Intron	1967–2094	1+9	0.01443 (0.00131)	0.01570 (0.00523)

Numbers of nucleotides in the second column correspond to the standard sequence, regardless indel variation in the natural population.

**Figure 4:**
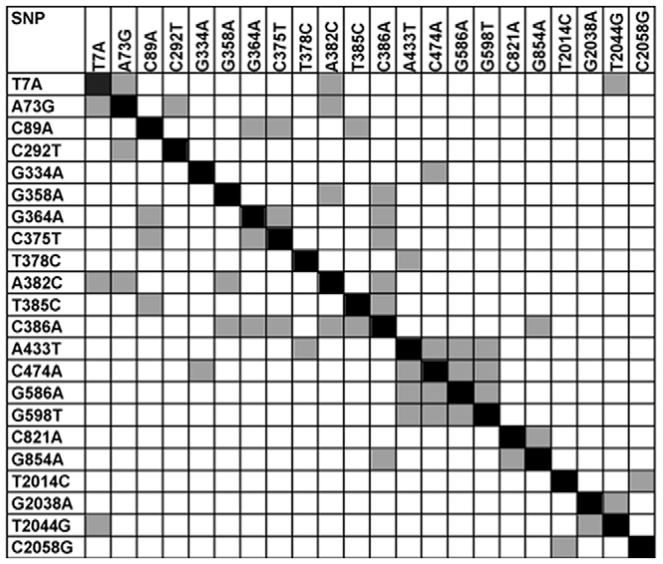
LD at the *Lim3* locus. Only markers with significant (corrected for multiple tests, DnaSP 4.0 [55]) LDs are included. LDs significant according both to Fishers's exact test and χ^2^ test are depicted in grey.

In total, 90 polymorphic markers were found, including 74 single nucleotide polymorphisms (SNPs) and 16 insertions and deletions (indels). Estimates of nucleotide diversity based on the number of differences between pairs of sites (π, [25]) and the number of segregating sites (θ, [26]) were within the range observed for *D. melanogaster* [27]: π = 0.00709±0.00046 and θ = 0.00864±0.00098. The highest level of variation was in the intron. However, when the regulatory region was divided arbitrarily into two equal parts, the distal section had approximately the same level of variation as the intron, while the proximal section closest to the 5′ UTR was much more conserved (Table 1). Not surprisingly, the most conserved was the translated part of the exon (Table 1), where only two nonsynonymous substitutions were found, each with a frequency of 0.02. Little significant linkage disequilibrium (LD) was observed between polymorphic markers (Figure 4), and the pattern of linked loci was as expected under assumptions of normal recombination, with few exceptions. This result was favorable for the identification of casual associations between molecular and phenotypic variations.

Molecular population genetic tests for selection were used to determine whether evolutionary forces might be regulating nucleotide variation at *Lim3* locus. Significant negative values for D [28], D* and F* [29] were observed for the first exon (Table 2). Most parameters were also significant for the 5′ UTR alone, and for the translated region of the exon alone (Table 2). Only two nonsynonymous polymorphisms were found in our sample, so other neutrality tests were not applied. Overall, our results indicated less variation in the *Lim3A* first exon than expected under neutral expectations, and the action of purifying selection on this region. To understand in more detail the biological significance of molecular variation observed in the Raleigh natural population, we tested effects of nucleotide diversity on gene expression and fly phenotype.

**Table 2:** Neutrality tests in the regulatory region and the beginning of the structural part of the *Lim3A*.

Region	D	D*	F*	D*, simulans	F*, simulans	D*, yakuba	F*, yakuba
All sequence	−0.63216	−1.75232	−1.59703	−1.99481	−1.89078		
Regulatory region	−0.31834	−1.03457	−0.92083	−1.35484	−1.28096		
	0.15386	−0.88538	−0.60895	−1.11283	−0.91096		
	−1.07509	−1.02695	−1.23886	−1.29958	−1.51835	−1.55057	−1.67083
Exon	**−2.10512**	**−3.73109**	**−3.79399**	**−3.40290**	**−3.56259**	**−2.78912**	**−3.07972**
5′UTR	**−2.04396**	**−3.26405**	**−3.37500**	**−2.78749**	**−3.02202**	−2.08900	**−2.46833**
Translated region	−1.46443	**−2.53305**	**−2.57464**	**−2.58770**	**−2.63091**	**−2.58770**	**−2.63091**
Intron	−0.22559	−1.28572	−1.10719	−0.44091	−0.35893	−1.99059	−1.81791

Significant D, D*, F* are in bold case (P<0.05 according to DnaSP 4.0 [55]), in bold case and underlined (P<0.02). Sequences from *D. simulans* and *D. yakuba* have GenBank accession no. XM_002079849 and NT_167063, respectively.

### Association between molecular variation at Lim3 locus and lifespan

Association studies used 44 polymorphic markers that were present in our sample at a frequency of 0.06 (in three lines out of 50) and higher. This restriction allowed us to concentrate on polymorphisms that were truly segregating in nature. Lifespan measurements were published in [5], [6].

Analysis of variance (ANOVA) revealed six polymorphic markers significantly associated with lifespan, while no significant association with lifespan was seen for sex or marker by sex interaction. Based on these results and the restricted sample size of sequenced alleles, we combined data on the sexes for nonparametric, distribution-free Wilcoxon tests, to assess association between molecular variation at *Lim3* and lifespan.

The same six markers showed significant association with lifespan (Figure 3, Table 3, Table S1): four were located in the regulatory region (A433T; G871C; A1050G; C1177T), one in the 5′ UTR (T1658C), and one in the first intron (G1991A). None were in significant LD with each other. We also checked association of lifespan with several haplotypes composed of combinations of significant markers from the regulatory region that were most likely to influence lifespan through transcription alteration. Haplotypes composed of four makers, A433T, G871C, A1050G, and C1177T, three proximal markers adjacent to the structural gene, G871C, A1050G, and C1177T, and two markers with minimal P-values for individual association with lifespan, G871C and C1177T, were significantly associated with lifespan (Table 3, Table S1). In total, we carried out 47 association tests. Only lifespan association with haplotype G871C+C1177T survived Bonferroni correction, and lifespan associations with two other haplotypes and the G1991A marker survived a less conservative false discovery rate (FDR) correction. We concluded that the combination of two markers in the regulatory region, G871C+C1177T, which were present in all haplotypes, and the single marker in the first intron of *Lim3A* were important for lifespan.

**Table 3:** Genotype-phenotype associations at the *Lim3* locus.

SNP, haplotype	Numbers of lines with alternative alleles	Trait	P value^3^	Mean (s. e.)
**A433T**	38/12^1^; 14/2^2^	Lifespan	*0.0357*	
		RNA, embryos	***0.0030***/0.1384	**0.7(0.06)/2.0(0.37)**
		RNA, heads	0.0682/0.1543	
		RNA, testes	*0.0325*/0.3327	
G586A, G598T	36/14^1^; 12/4^2^	Lifespan	0.0992	
		RNA, embryos	***0.0131***/0.0896	**0.7(0.06)/1.4(0.29)**
		RNA, heads	0.1773/0.2230	
		RNA, testes	*0.0198*/0.0896	
**G871C**	46/4^1^; 13/3^2^	Lifespan	*0.0151*	
		RNA, embryos	***0.0002***/***0.0059***	**0.6(0.06)/1.8(0.27)**
		RNA, heads	***0.0105***/*0.0180*	**0.4(0.05)/0.7(0.12)**
		RNA, testes	*0.0138*/0.1924	
A926G	47/3^1^; 14/2^2^	Lifespan	0.0891	
		RNA, embryos	***0.0021***/0.0682	**0.7(0.06)/2.0(0.37)**
		RNA, heads	***0.0052***/***0.0025***	**0.4(0.05)/0.8(0.07)**
		RNA, testes	*0.0209*/0.4588	
G1021A,	40/10^1^; 10/6^2^,	Lifespan	0.7528, 0.6801	
C1046A	39/11^1^; 10/6^2^	RNA, embryos	***0.0018***/*0.0195*	**1.1(0.20)/0.5(0.09)**
		RNA, heads	*0.0356*/***0.0091***	
		RNA, testes	*0.0091*/***0.0014***	
**A1050G**	26/24^1^; 11/5^2^	Lifespan	*0.0226*	
		RNA, embryos	0.5153/0.8548	
		RNA, heads	0.9029/0.2550	
		RNA, testes	0.9190/0.1039	
**C1177T**	47/3^1^; 14/2^2^	Lifespan	*0.0084*	
		RNA, embryos	***0.0033***/***0.0037***	**0.9(0.11)/0.3(0.04)**
		RNA, heads	***0.0021***/***0.0021***	**0.5(0.05)/0.1(0.02)**
		RNA, testes	*0.0121*/*0.0044*	
**G1991A**	46/4^1^; 13/3^2^	Lifespan	***0.0028***	**37(1)/29(2)**
		RNA, embryos	0.2992/0.2005	
		RNA, heads	0.2183/0.2274	
		RNA, testes	0.4115/0.2333	
**T1658C**	45/5^1^; 13/3^2^	Lifespan	*0.0195*	
		RNA, embryos	0.1842/0.2881	
		RNA, heads	0.1111/0.1475	
		RNA, testes	0.1407/0.0535	
**871+1177,**	4/43/3^1^; 3/11/2^2^	Lifespan	***0.0010***	**31(2)/38(1)/29(2)**
**CC/GC/GT**		RNA, embryos	***0.0001***/***0.0014***	**1.8(0.27)/0.7(0.06)/0.3(0.04)**
		RNA, heads	***0.0011***/***0.0016***	**0.7(0.12)/0.4(0.04)/0.1(0.02)**
		RNA, testes	*0.0053*/*0.0125*	

1 Data for the sample of 50 lines.

2 Data for the sample of 16 lines.

3 For associations with lifespan, P values of Wilkoxon test of line means, and for associations with *Lim3* transcription, P values of Wilkoxon test of mRNA amounts/C(t) are shown, see text for details.

Significant P values are in italics; P values surviving FDR correction are in italics and bold case; P values surviving Bonferroni correction are in italics, bold case and underlined.

Markers and haplotypes significantly associated with lifespan are in bold case.

One of the alleles at each polymorphic site composing the significant haplotype had a low population frequency (p_C_ = 0.08 for C871G; p_T_ = 0.06 for C1177T), and was associated with short lifespan (Table 3). Of four possible combinations of alleles, only three were present in the population. Their frequencies were in good agreement with those expected from the frequencies of single alleles (χ^2^ = 0.0055), which confirmed the absence of LD between the markers. Multiple comparisons of means allowed us to divide the GC, CC, and GT haplotype variants of the G871C and C1177T markers into two groups that significantly differed in lifespan (P<0.05). The first group included 86% of lines and was characterized by the GC haplotype and a mean lifespan of 38 (±1) days. The second group included lines with the rare CC (8%) and GT (6%) haplotype, and mean lifespans of 31 (±2) and 29 (±2) days.

We proposed that polymorphisms in the regulatory region of the gene affect its expression, and thus a phenotypic trait such as lifespan. Our next goal was to test this hypothesis experimentally.

### Association between molecular variation in Lim3A regulatory region and Lim3A expression

*Lim3A* and *Lim3C* differ in their 5′ UTR region, with *Lim3C* shorter by 190 bp. Therefore, the amount of either *Lim3A* alone, or both transcripts could be detected and measured. As *Lim3A* was more abundant (Figure 2A), and has functional significance for neuron development [10], we focused our analysis on *Lim3A*. To assess association between molecular variation in the *Lim3A* regulatory region and its transcript level, 16 lines with different G871C and C1177T haplotypes were selected. According to the information available [http://flyatlas.org, accession no. FBgn0002023], *Lim3* transcription is predominantly observed in embryos, and in adult brains and testes. Guided by this information, we evaluated the amount of *Lim3A* in embryos, heads (Table 4), and testes of selected lines using real time RT-PCR.

**Table 4:** Polymorphism in the TFs binding sites and *Lim3A* transcription.

Line	Nucleotide in the putative binding site for Grh	Nucleotide in the putative binding site for CA/TG – repeat binding protein	*Lim3A* mRNA amount in embryos (s. e.)	*Lim3A* mRNA amount in heads (s. e.)	Mean lifespan, males and females combined from [5], days
284	C	***C***	2.578 (0.241)	0.910 (0.078)	27.5
207	C	***C***	1.369 (0.116)	0.774 (0.195)	41.5
285	C	***C***	1.361 (0.119)	0.514 (0.077)	27.5
472	***G***	***C***	1.068 (0.364)	0.232 (0.027)	37.0
74	***G***	***C***	1.045 (0.043)	0.301 (0.067)	38.0
100	***G***	***C***	0.876 (0.097)	0.432 (0.061)	48.5
40	***G***	***C***	0.847 (0.028)	0.402 (0.059)	51,0
115	***G***	***C***	0.729 (0.085)	0.490 (0.034)	51.5
201	***G***	***C***	0.676 (0.033)	0.757 (0.085)	30.0
325	***G***	***C***	0.615 (0.148)	0.712 (0.057)	42.5
336	***G***	***C***	0.481 (0.130)	0.420 (0.144)	40.0
200	***G***	***C***	0.457 (0.138)	0.349 (0.009)	36.5
461	***G***	***C***	0.452 (0.043)	0.394 (0.022)	30.5
180	***G***	***C***	0.439 (0.134)	0.193 (0.022)	46.5
76	***G***	T	0.358 (0.039)	0.127 (0.005)	31.0
226	***G***	T	0.236 (0.049)	0.161 (0.049)	23.5

Alleles corresponding to the standard sequence are in italics and bold case.

Correlations between independent measurements of *Lim3A* transcripts were highly significant across the 16 lines in both embryos (P<0.0001) and in heads (P = 0.0074), strengthening reliability of the results. The correlation between independent measurements in the testes was not significant (P = 0.1082), probably because of the substantially smaller amount of detected *Lim3A* mRNA. The amount of *Lim3A* mRNA was also correlated in embryos and heads (P = 0.0064), in embryos and testes (P = 0.0579), and in heads and testes (P = 0.0204) across the 16 lines.

In total, 30 of the 44 markers segregated in these lines, and eight were in complete LD with the others: 24 association tests with 22 markers and two haplotypes were performed. According to the distribution-free Wilcoxon test, significant association was seen between *Lim3A* levels in embryos for 14 polymorphic markers. For four markers (G871C, A926G, G1021A, C1046A), this held after Bonferroni correction, and another four (A433T, G586A, G598T, C1177T) held after FDR correction (Table 3, Table S1). Markers G1021A and C1046A, G586A and G598T were in complete LD in the 16 lines. Another method [30] based on the analysis of direct C(t) measurements proportional to the logarithm of the substrate quantity was used for verification. Significant associations surviving FDR corrections were confirmed for G871C and C1177T (Table 3, Table S1). Finally, REST [31], a program that accounts for different PCR efficiencies for target and reference genes, confirmed associations of G871C and C1177T (P = 0.0001 for both).

**Figure 5:**
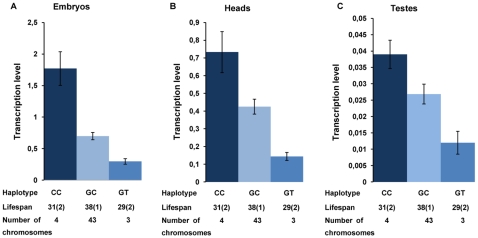
*Lim3A* transcription level in lines with different haplotype variants. (A). *Lim3A* transcription level in embryos. (B). *Lim3A* transcription level in adult heads. (C). *Lim3A* transcription level in adult testes. Haplotypes composed of segregating markers G871C and C1177T, haplotype mean lifespan in days (s. e.) and numbers of chromosomes with corresponding haplotypes sampled from Raleigh population are given below the diagrams.

G871C and C1177T are the two polymorphic markers that form the haplotype that is significantly associated with lifespan. Association with *Lim3A* levels in embryos was highly significant for this haplotype, by all methods of analysis (Table 3), including pairwise comparisons using REST software (P = 0.0001 for each comparison). Multiple comparisons of means allowed us to categorize lines with different haplotype variants of the G871C and C1177T markers, specifically CC, GC, and GT, into three groups with an approximately six-fold significant difference (P<0.05) in the amount of *Lim3A* in embryos (CC: 1.8±0.27; GC: 0.7±0.06; GT: 0.3±0.04; Figure 5).

According to the Wilcoxon test, significant associations were found for *Lim3A* levels in adult heads for five polymorphic markers. One marker (C1177T) survived Bonferroni correction and another two (G871C, A926G) survived FDR correction (Table 3, Table S1). Analysis of direct C(t) measurements revealed significant associations surviving FDR correction for A926G and C1177T (Table 3, Table S1). These results were not confirmed using REST. Association with *Lim3A* levels in adult heads was highly significant for the G871C+C1177T haplotype by both nonparametric analysis methods (Table 3), and only one of the three pair-wise comparisons was significant (REST, P = 0.023 for GT compared to CC). Multiple comparisons of means allowed us to categorize lines with the CC, GC, and GT haplotype variants of the G871C and C1177T markers, into three groups with approximately six-fold significant differences (P<0.05) in *Lim3A* levels in adult heads (CC: 0.7±0.12; GC: 0.4±0.04; GT: 0.1±0.02; Figure 5).

According to the Wilcoxon test, significant associations were seen between the amount of *Lim3A* in testes and 16 polymorphic markers, although none survived Bonferroni or FDR correction (Table 3, Table S1). Association was also significant for the G871C+C1177T haplotype, according to both nonparametric analysis methods, but these also did not survive Bonferroni or FDR correction (Table 3). Multiple comparisons of means showed that *Lim3A* transcription in testes was significantly different (P<0.05) between lines with the CC (0.04±0.004) and GT (0.01±0.003) haplotype variants of the G871C and C1177T markers (Figure 5).

Many polymorphic markers appeared to be significantly associated with the amount of *Lim3A* in different tissues. Different methods of analysis and different P-value corrections gave slightly different, though not contradictory results (Table 3). The most notable polymorphic markers were G871C and C1177T, which formed a haplotype significantly associated with lifespan (P = 0.0010), and with transcription in embryos (P = 0.0001), adult heads (P = 0.0011), and testes (P = 0.0053). Each of the two markers alone was also significantly associated with transcription in embryos (G871C: P = 0.0002; C1177T: P = 0.0033), adult heads (P = 0.0105, P = 0.0021), and testes (P = 0.0138, P = 0.0121), as well as lifespan (P = 0.0151, P = 0.0084). The polymorphic markers A926G and G1021A+C1046A (linked in the sample of 16 lines), located between G871C and C1177T, were also significantly associated with the *Lim3* transcription level in embryos (A926G: P = 0.0021; G1021A+C1046A: P = 0.0018), adult heads (P = 0.0052, P = 0.0356), and testes (P = 0.0209, P = 0.0091), as well as the haplotype composed of all five markers G871C+A926G+(G1021A+C1046A)+C1177T (P = 0.0005, P = 0.0058, P = 0.0053, for embryos, heads and testes, Table S1). We propose that the entire region from 380 to 686 bp upstream of the *Lim3A* major TSS is important for gene expression, while only two markers within this region are important for lifespan.

All five polymorphic markers mentioned above are in potential TF-binding sites: G871C and A926G are in the Grainy Head (Grh) binding site consensus sequence, G1021A is in the specificity protein-1 (Sp1)/Krüppel-like factor (KLF) binding-site consensus sequence, C1046A in the Zeste-like motif, and C1177T is in the (CA/TG)_9_ repeat (Figure 3).

G871C and C1177T appeared to be the most essential markers for *Drosophila Lim3A* expression and lifespan. When C, a frequent allele in the Raleigh population, was present at the 1177 position, *Lim3A* transcription was intermediate and *Drosophila* lifespan was high. When C was substituted for T, a rare allele, the expression and *Drosophila* lifespan were low (Table 4). Hence, we suggest that this site normally functions in *Lim3* activation, as an activator-binding site. The (CA/TG)_9_ repeat where the C1177T polymorphic site is located is a a cis-regulatory element [32], however, nothing is known about the proteins that bind this repeat [33].

In a background of C at the 1177 position, *Lim3A* transcription and *Drosophila* lifespan was dependent on the G871C marker (Table 4). When G, a frequent allele in Raleigh population, was present at the 871 position, *Lim3A* transcription was intermediate and *Drosophila* lifespan was high. When G was substituted for C, a rare allele, the expression increased, and *Drosophila* lifespan was short (Table 4). Hence, we suggest that normally this site is involved in *Lim3* repression as a repressor binding site. Indeed, Grh, which presumably interacts with G871C as part of its specific binding site, cooperates with Polycomb-group (PcG) proteins that inactivate genes by chromatin remodeling, and Grh-binding sites are often encountered in Polycomb response elements (PREs) [34]–[36].

Both intermediate level of *Lim3A* expression and longer lifespan are associated with the same polymorphic haplotype. Square regression (with mean lifespan as a dependent variable and *Lim3A* mRNA amount as independent variable) is a better approximation for our data (R^2^ = 0.062 for embryos; R^2^ = 0.011 for heads; R^2^ = 0.007 for testes) than linear regression (R^2^ = 0.0029 for embryos; R^2^ = 0.0006 for heads; R^2^ = 0.000 for testes). For embryos, square regression is significant (P = 0.0472), indicating that the model accounts for a low but significant portion of variation in the data; linear regression is not significant (P = 0.2814). This result is in agreement with the hypothesis that intermediate levels of *Lim3A* expression confer longer lifespan.

## Discussion

We found that *Lim3* produces three mRNAs. In addition to the already known *Lim3A* and *Lim3B* transcript, we discovered the additional *Lim3C* mRNA. The promoter region of *Lim3A* is DPE-containing, but lacks a TATA box, and possesses multiple start sites with one major initiation site, and additional nearby minor ones. The distance between the *Lim3A* DPE and initiator is appropriate for TFIID binding, which is essential for transcription [37]. Reduced expression of *Lim3C* compared to *Lim3A* is most likely explained by the lack of a strong initiator, TATA-box, or other core promoter elements. However, other elements such as CAAAAT, and other different mechanisms of initiation may be used to regulate *Lim3C* transcription. The alternative promoters of *Lim3A* and *Lim3C* may provide a mechanism for tissue- and developmental stage-specific *Lim3* activation. TATA box-containing promoters are activated after embryonic development, and TATA-less promoters of the same genes are active during early embryo development [38], [39]. Mammalian *LHX3a* and *LHX3b*, which are homologues of *Lim3A* and *Lim3B*, are transcribed from two alternative TATA-less, GC-rich promoters [40], have distinct temporal expression profiles, and have different regulatory roles in the development of the distinct cell types [41].

Statistical analyses demonstrated that the first exon of *Lim3A* (*Lim3C*) is affected by purifying selection. The normal recombination found in this region suggests that the selection should be highly effective against deleterious alleles, removing them from the population [42]. Indeed, only two polymorphisms with minimal detectable frequency were found in the translated region of the first exon. Thus, the conserved structure of the Lim3A (Lim3C) protein can be assumed to be essential for its proper function, and therefore maintained by selection. An alternative explanation is that the Raleigh population recently experienced a bottleneck. However, this is not confirmed by analysis of selection forces acting on other regions of the gene, or on other genes whose molecular variation was analyzed using the same sample of second chromosomes from the Raleigh population (*Dopa dcarboxilase* [5]; *Catecholamines Up* [6]; *shuttle craft*, Simonenko, Pasyukova, unpublished results).

Regulatory regions can have crucial roles in evolution, and modifications in these regions have mainly adaptive evolutionary effects [43], [44]. Statistical analysis did not reveal any evidence for natural selection in the *Lim3A* regulatory region. Nevertheless, the significance of the regulatory region for transcription and phenotype was demonstrated by the finding that nucleotide substitutions within this region that segregated in the Raleigh population appeared to result in differences as large as six-fold in gene transcription, and 1.3-to-1.5-fold in lifespan. No significant associations were found between markers located outside the regulatory region, (*i.e.* in the 5′ UTR or the *Lim3A* structural gene) and *Lim3A* levels, and markers significantly associated with *Lim3A* expression were not in LD with each other or with markers within the gene in a sample of 50 alleles. Therefore, we have likely identified actual casual relationships between natural polymorphisms and gene function. Haplotype variants of the G871C and C1177T polymorphic markers associated with short lifespan, and either high or low *Lim3A* transcription (CC, GT) were found in the Raleigh population at low frequencies. The haplotype variant associated with long lifespan and intermediate *Lim3A* transcription (GC) was present at high frequency. Thus, association analysis predicted that an intermediate level of *Lim3A* expression provided longer lifespan, and a selective advantage. Statistical tests were possibly not sensitive enough to detect this selection, however. Even when the fitness effects of mutations are in the nearly neutral range, natural selection is still able to influence transcriptional phenotype [45].

A possible general explanation for the absence of selection on the regulatory region is that nucleotide substitutions in a single, or in several TF binding sites might affect gene expression only in the tissues where these TFs are active, so the impact of the substitutions on phenotypic traits would be small. However, as mentioned above, this was not true for several polymorphisms within the *Lim3A* 5′-regulatory region, which significantly affected expression and phenotype. Moreover, it is difficult to point to polymorphic markers within *Lim3A* regulatory region which have tissue-specific effects. Rather, most polymorphic markers that were significantly associated with transcript abundance seemed to be important in all tissues, and the exact significance level of the effect depended on the reliability of measurements in a particular tissue and on methods of analysis. Thus, nucleotide substitutions found in the *Lim3A* regulatory region in the Raleigh natural population must be located within sites that regulate transcription in a general, rather than a tissue-specific manner.

Most polymorphic markers significantly associated with transcription were located in the compact region that was 380–680 bps upstream of the *Lim3A* major TSS, and were within binding sites for important transcriptional regulators. For example, Grh is involved in many regulatory networks, including the complex regulation of neuroblast specification and neuron apoptosis [46], [47]. Sp1 mediates transcription of the *LHX3* gene, the human homologue of *Lim3* [40]. Grh and Sp1/KLF are members of the PcG and trxG complexes. Binding sites for other members of these complexes (Pho, GAGA or GAF/Psq), were also found in the *Lim3A* regulatory region, suggesting that PRE-TRE sites for PcG and trxG complexes are present in the region.

We presume that both repressor and activator proteins bind the essential sites for *Lim3* transcription and fly lifespan in which the polymorphic markers are located. We hypothesize that the repressor protein Grh and the unknown activator protein that binds the (CA/TG)_9_ repeat might provide negative and positive transcriptional regulation of *Lim3A*, and consequently affect *Drosophila* lifespan. Disrupting the balance between negative and positive regulation would result in deviations in *Lim3A* transcription, and a decrease in D*rosophila* lifespan An intermediate expression based on a balance between activation and repression of the gene and favorable for long lifespan could be provided by the combined activity of PcG and trxG protein complexes through maintenance of a silent or active transcriptional state of their target genes. The PcG and trxG complexes bind to genes encoding transcription factors, including homeodomain-containing proteins such as *Lim*3, and are implicated in the regulation of various transcriptional pathways [48].

Overexpression or RNAi knockdown of a number of *Drosophila* genes showed the involvement of these genes in lifespan control [for example, 49–51]. Direct proof of Lim3 involvement in lifespan control is required, however, gene overexpression or RNAi knockdown are not applicable in this particular case. We are considering site-specific integration of a *Lim3* transgene using carefully chosen sets of landing sites, transgene constructs, and drivers as a possible approach to verify the results presented here. Experimental manipulations with *Lim3* expression levels are also necessary to prove that intermediate levels of *Lim3A* expression confer longer lifespan.

The mechanism underlying D*rosophila* lifespan variation through alteration of *Lim3* expression is not understood. Molecular variation at the *Lim3* regulatory region most strongly affected *Lim3* expression in embryos. Previously, *Lim3* was found to be active in the *Drosophila* embryonic nervous system and to take part in regulatory networks leading to the specification of motor neuron subclass identity, axon pathfinding, and finally, proper muscle innervation [12]. *Lim3* was reported to be expressed in the *Drosophila* ring gland [10], but later studies failed to confirm *Lim3* expression in the embryonic *Drosophila* endocrine system [52]. Whether these *Lim3* functions are sufficient to explain the lifespan variations caused by alterations in *Lim3* expression in embryos, and other mechanisms that might explain lifespan effects initiated during early development are unknown. Recently, however, genes responsible for sex determination during early *Drosophila* development that also affect lifespan were found [53].

The role of *Lim3* in adult flies is not known, and we do not possess any information about alterations of *Lim3* transcription level with age. *Lim3* was first discovered as a male-specific candidate lifespan gene [8]. Thus, *Lim3* expression in testes is assumed to affect lifespan and even to have a main casual relation to lifespan variation. However, we failed to find a strong association between *Lim3* transcription in testes and lifespan, probably because of insufficient sensitivity in measuring of small amounts of mRNA. We demonstrated that *Lim3* is substantially expressed in adult heads. This confirms that *Lim3* expression in adults is tissue-specific, and probably associated with the nervous system. *Lim3* function in the adult brain may be involved in lifespan regulation. We intend to ascertain the *Lim3* function in the nervous and neuroendocrine systems of adult flies, to move closer to understanding the mechanisms underlying *Lim3* involvement in *Drosophila* lifespan control.

## Materials and Methods

### Drosophila stocks

We used 50 substitution *D. melanogaster* lines containing second chromosomes from the Raleigh (USA) population in homozygous Samarkand genetic background and differing in lifespan (22–62 days, P<0, 0001; [5]). All lines were reared in glass vials with wheat-sugar-agar medium, at 25°C.

### Nucleic acids isolation

DNA was extracted from 50 lines according to the standard procedures [54]. Total RNA for Northern and 5′RACE analyses was isolated using the SV Total RNA Isolation System (Promega) according to the manufacturer's instructions. Total RNA for real-time quantitative PCR was extracted from 50 12-hour embryos and from 20 heads (10 males and 10 females) or 50 pairs of testes of 15-day old adult flies using Trizol reagent (Invitrogen) and DNase I Kit (TURBO DNA-*free*™, Ambion) according to the manufacturers' instructions.

### DNA sequencing and analysis

Isolated DNA was used in PCR reaction with forward primer TCC AAC CAG ACT GTC AAG TCA AAT TAC and reverse primer TTG CAG AAA GAG AAT AAC GCT AAA TCA. Then PCR products were sequenced with Big Dye Terminator V. 3.1. Kit (Applied Biosystems), according to the manufacturer's protocol, on ABI PRIZM 310 Genetic Analyser (Applied Biosystems). Six primers for sequencing were used: TCC AAC CAG ACT GTC AAG TCA AAT TAC, TTC AAT TAG CAT GAT CCA AGG, AGA CGT TGC TCT CGA TCA GC, AGA CCA CTC CCC GAA AAC TG, ATG TCA GCC CCT GTA TGT GC, AGA ATC CAA TCA GAG TGC GTC.

The putative RNA Polymerase II promoter sites were predicted with Neural Network Promoter Prediction database, version 2.2 (http://www.fruitfly.org/seq_tools/promoter.html), Eukaryotic Promoter Database Current Release 100 (http://www.epd.isb-sib.ch/seq_download.html) and the Drosophila core promoter database (http://www.biology.ucsd.edu/labs/kadonaga/DCPD). To identify possible binding sites of transcription factors TFSEARCH at http://www.cbrc.jp/research/db/TFSEARCH.htm; MOTIF Search at http://motif.genome.jp/; ConSite at http://asp.ii.uib.no:8090/cgi-bin/CONSITE/consite/; TESS at http://www.cbil.upenn.edu/cgi-bin/tess/tess; Match™ at http://www.gene-regulation.com/cgi-bin/pub/programs/match/bin/match.cgi; Drosophila Melanogaster Major Position Matrix Motifs at http://line.imb.ac.ru/DMMPMM/ were used.

### Northern blot

Total RNA was quantified by absorbance at 260 nm, and 8–14 mg of total RNA were resolved by 1.5% denaturing agarose-formaldehyde gel, blotted onto Hybond-N+ membrane (Amersham) and fixed by UV cross-linking. Pre-hybridization and hybridization were performed at 42°C overnight in 25ml of the solution containing 50% formamide, 10× Denhardt solution, 5×SSC, 0.5% SDS, 100mg/ml tRNA. After hybridization, membrane was washed twice (10 min each) in 2×SSC, 0.1% SDS at 42°C, three times (5 min each) in 0.1×SSC, 0.1% SDS at 55°C, and exposed on a phosphor-imager (Storm, Amersham) using Image Quant Version 5.2 computational tool.

A 1640 bp *Lim3* PCR fragment was amplified (forward primer: TTC AAT TAG CAT GAT CCA AGG, reverse primer: TCA CAT TTG CCA TTG GAC AGG AAG TC) and used as a probe to detect *Lim3* transcripts. DNA probes (5–10×10^6^ cpm) added to the hybridization mixture were labeled by Hexa Label™ DNA Labeling Kit (Fermentas) with 20–40 µCi [α-^32^P] and then purified with CentriSep columns (Princeton Separations).

### Rapid amplification of 5′cDNA end (5′RACE) analysis

Transcription start sites of *Lim3A* mRNA in *D. melanogaster* were identified with the rapid amplification of the cDNA ends (RACE) technique using Smart™ RACE cDNA Amplification Kit (Clontech) for the first-strand cDNA synthesis. The touchdown PCR of the first-strand cDNA was then performed by using the gene-specific reverse primer, TCA CAT TTG CCA TTG GAC AGG AAG TC and the manufacturer's Abridged Anchor primer (Smart™ RACE cDNA Amplification Kit, Advantage 2 Polymerase Mix, Clontech). The annealing was performed at 64°C for 30 sec and extension at 68°C for 3 min, other parameters of the touchdown PCR were selected according to the manufacturer's recommendations. PCR products were gel-purified (Wizard® PCR Preps DNA Purification System, Promega) and cloned into pGEM-T EasyVector (Promega). Plasmid DNA was isolated (Wizard® *Plus* Minipreps DNA Purification Systems, Promega) and sequenced.

### Real-time RT-PCR

The first strand of cDNA was synthesized using Super Script™ II Reverse Transcriptase (Invitrogen) with oligo(dT) primer, according to the manufacturer's instructions. cDNA amount was analyzed by real-time quantitative PCR using SYBR Green I/Rox in Chromo4 Real-Time PCR Detector (Bio-Rad). Equal amounts of mRNA and cDNA for real time RT-PCR analysis were used to evaluate the *Lim3A* expression in various tissues and life stages.

*Gdh*, a housekeeping gene located on the chromosome 3 which was common to all the substitution lines and characterized by relatively low expression level comparable with expression level of *Lim3* was used as a reference gene to normalize for differences in total cDNA between samples. Forward and reverse primer sequences were: *Lim3-RA*: TGT GAA AGT GAT GGT TGA TTG CTC TGC, TCA CAT TTG CCA TTG GAC AGG AAG TC; *Gdh*: TAT GCC ACC GAG CAC CAG ATT CC, GGA TGC CCT TCA CCT TCT GCT TCT T.

MJ Opticon Monitor™ Analysis Software V. 3.1. 32 (Bio-Rad laboratories Inc., 2004–2005) was used to evaluate C(t) value and relative *Lim3A* mRNA amount which was considered as a measure of *Lim3* transcription level in each *Drosophila* line.

### Statistical analyses

The nucleotide diversity was analyzed as the pairwise distance between alleles (π) and the average number of segregating sites (θ) using DnaSP 4.0 [55]. This software was also used to assess linkage disequilibrium (LD) between polymorphic sites, and selective neutrality of observed polymorphisms (D, D* and F*, D* and F* with outgroup, [28], [29]).

Association between molecular polymorphisms and lifespan was assessed by two-way fixed effects ANOVA of line means, with polymorphic marker and sex as main effects, and by nonparametric distribution free Wilkoxon test of line means. Association between molecular polymorphisms and *Lim3* transcription was assessed by nonparametric distribution free Wilkoxon test of mRNA amount or C(t)s [30]. REST V2.0.7 program [31], with the number of randomizations equal to 10,000, was used to verify the results. Multiple comparison of means (Tukey's test) was used to compare lifespan and *Lim3* expression in groups of lines with different molecular haplotypes. Regression analysis with mean lifespan as a dependent variable and *Lim3A* mRNA amount as independent variable was used to assess association between lifespan and *Lim3* transcription. Bonferroni and False Discovery Rate (FDR, [56]) corrections for multiple analyses were used when appropriate.

## Supporting Information

Table S1 Genotype-phenotype associations at the Lim3 locus.
(0.17 MB DOC)
