# Supplementary material for: A Naturally Occurring Polymorphism at Drosophila melanogaster Lim3 Locus, a Homolog of Human LHX3/4, Affects Lim3 Transcription and Fly Lifespan
Source: PLoS One. 2010 Sep 7;5(9):e12621. doi: 10.1371/journal.pone.0012621 (PMC2935391; doi:10.1371/journal.pone.0012621)
Supplement: Table S1 — Genotype-phenotype associations at the Lim3 locus. (0.17 MB DOC) [file pone.0012621.s001.doc]

Table S1. Genotype-phenotype associations at the *Lim3* locus

| SNP, haplotype | Numbers of lines with alternative alleles | Trait | P value^4^ |
| --- | --- | --- | --- |
| T7A | 37/13^1^; 11/5^2^ | Lifespan | 0.3677 |
|  |  | RNA, embryos | 0.5285/0.7759 |
|  |  | RNA, heads | 0.4895/0.7450 |
|  |  | RNA, testes | 0.1431/*0.0184* |
| G46A,C | 46/3/1^3^ | Lifespan | 0.9899 |
|  |  | RNA, embryos | - |
|  |  | RNA, heads | - |
|  |  | RNA, testes | - |
| A73G | 35/15^1^; 10/6^2^ | Lifespan | 0.5394 |
|  |  | RNA, embryos | 0.1498/0.5592 |
|  |  | RNA, heads | 0.6685/0.3918 |
|  |  | RNA, testes | *0.0292*/*0.0111* |
| C89A | 31/19^1^; 9/7^2^ | Lifespan | 0.3361 |
|  |  | RNA, embryos | 0.8792/0.8493 |
|  |  | RNA, heads | 0.9697/0.4941 |
|  |  | RNA, testes | 0.5431/*0.0367* |
| C151G | 45/5^3^ | Lifespan | 0.9725 |
|  |  | RNA, embryos | - |
|  |  | RNA, heads | - |
|  |  | RNA, testes | - |
| G156A | 47/3^3^ | Lifespan | 0.3873 |
|  |  | RNA, embryos | - |
|  |  | RNA, heads | - |
|  |  | RNA, testes | - |
| G164A | 47/3^3^ | Lifespan | 0.3873 |
|  |  | RNA, embryos | - |
|  |  | RNA, heads | - |
|  |  | RNA, testes | - |
| C292T | 33/17^1^; 9/7^2^ | Lifespan | 0.6212 |
|  |  | RNA, embryos | *0.0334*/0.2389 |
|  |  | RNA, heads | 0.2100/0.1024 |
|  |  | RNA, testes | *0.0420*/*0.0062* |
| C316T | 46/4^3^ | Lifespan | 0.3906 |
|  |  | RNA, embryos | - |
|  |  | RNA, heads | - |
|  |  | RNA, testes | - |
| G334A | 44/6^3^ | Lifespan | 0.4509 |
|  |  | RNA, embryos | - |
|  |  | RNA, heads | - |
|  |  | RNA, testes | - |
| A346T | 46/4^3^ | Lifespan | 0.8437 |
|  |  | RNA, embryos | - |
|  |  | RNA, heads | - |
|  |  | RNA, testes | - |
| G358A | 37/13^1^; 9/7^2^ | Lifespan | 0.7556 |
|  |  | RNA, embryos | *0.0334*/0.2389 |
|  |  | RNA, heads | 0.2100/0.1024 |
|  |  | RNA, testes | *0.0420*/*0.0062* |
| G364A | 26/24^1^; 8/8^2^ | Lifespan | 0.1399 |
|  |  | RNA, embryos | 0.2000/0.7630 |
|  |  | RNA, heads | 0.2744/0.0765 |
|  |  | RNA, testes | 0.1049/*0.0288* |
| C375T | 28/22^1^; 9/7^2^ | Lifespan | 0.6464 |
|  |  | RNA, embryos | 0.2241/0.9546 |
|  |  | RNA, heads | 0.3233/0.0806 |
|  |  | RNA, testes | 0.1104/*0.0135* |
| T378C | 39/11^3^ | Lifespan | 0.6831 |
|  |  | RNA, embryos | - |
|  |  | RNA, heads | - |
|  |  | RNA, testes | - |
| A382C | 33/17^1^; 9/7^2^ | Lifespan | 0.9651 |
|  |  | RNA, embryos | *0.0334*/0.2389 |
|  |  | RNA, heads | 0.2100/0.1024 |
|  |  | RNA, testes | *0.0420*/*0.0062* |
| T385C |  | Lifespan |  |
|  |  | RNA, embryos |  |
|  |  | RNA, heads |  |
|  |  | RNA, testes |  |
| C386A |  | Lifespan |  |
|  |  | RNA, embryos |  |
|  |  | RNA, heads |  |
|  |  | RNA, testes |  |
| **A433T** | 38/12^1^; 14/2^2^ | Lifespan | *0.0357* |
|  |  | RNA, embryos | ***0.0030***/0.1384 |
|  |  | RNA, heads | 0.0682/0.1543 |
|  |  | RNA, testes | *0.0325*/0.3327 |
| T449G,C | 40/9/1^3^ | Lifespan | 0.1399 |
|  |  | RNA, embryos | - |
|  |  | RNA, heads | - |
|  |  | RNA, testes | - |
| C474A | 42/8^3^ | Lifespan | 0.4979 |
|  |  | RNA, embryos | - |
|  |  | RNA, heads | - |
|  |  | RNA, testes | - |
| -491TAAACTTCTCA | 40/10^3^ | Lifespan | 0.1311 |
|  |  | RNA, embryos | - |
|  |  | RNA, heads | - |
|  |  | RNA, testes | - |
| G586A | 36/14^1^; 12/4^2^ | Lifespan | 0.0992 |
|  |  | RNA, embryos | ***0.0131***/0.0896 |
|  |  | RNA, heads | 0.1773/0.2230 |
|  |  | RNA, testes | *0.0198*/0.0896 |
| G598T | 36/14^1^; 12/4^2^ | Lifespan | 0.0992 |
|  |  | RNA, embryos | ***0.0131***/0.0896 |
|  |  | RNA, heads | 0.1773/0.2230 |
|  |  | RNA, testes | *0.0198*/0.0896 |
| G631T | 41/9^1^; 11/5^2^ | Lifespan | 0.6631 |
|  |  | RNA, embryos | 0.5153/0.8548 |
|  |  | RNA, heads | 0.9029/0.2550 |
|  |  | RNA, testes | 0.9190/0.1039 |
| T643G | 40/10^1^; 12/4^2^ | Lifespan | 0.3559 |
|  |  | RNA, embryos | 0.2148/0.6922 |
|  |  | RNA, heads | 1.0000/0.5716 |
|  |  | RNA, testes | 0.3166/0.1637 |
| C821A | 34/16^1^; 9/7^2^ | Lifespan | 0.9734 |
|  |  | RNA, embryos | *0.0276*/0.1598 |
|  |  | RNA, heads | 0.0575/***0.0070*** |
|  |  | RNA, testes | *0.0318*/*0.0098* |
| CA845- | 35/15^1^; 9/7^2^ | Lifespan | 0.9071 |
|  |  | RNA, embryos | *0.0276*/0.1598 |
|  |  | RNA, heads | 0.0575/***0.0070*** |
|  |  | RNA, testes | *0.0318*/*0.0098* |
| G854A | 34/16^1^; 8/8^2^ | Lifespan | 0.6571 |
|  |  | RNA, embryos | 0.3271/0.2912 |
|  |  | RNA, heads | 0.8505/0.5977 |
|  |  | RNA, testes | 0.3176/*0.0093* |
| **G871C** | 46/4^1^; 13/3^2^ | Lifespan | *0.0151* |
|  |  | RNA, embryos | ***0.0002***/***0.0059*** |
|  |  | RNA, heads | ***0.0105***/*0.0180* |
|  |  | RNA, testes | *0.0138*/0.1924 |
| A926G | 47/3^1^; 14/2^2^ | Lifespan | 0.0891 |
|  |  | RNA, embryos | ***0.0021***/0.0682 |
|  |  | RNA, heads | ***0.0052***/***0.0025*** |
|  |  | RNA, testes | *0.0209*/0.4588 |
| G1021A | 40/10^1^; 10/6^2^, | Lifespan | 0.7528, |
|  |  | RNA, embryos | ***0.0018***/*0.0195* |
|  |  | RNA, heads | *0.0356*/***0.0091*** |
|  |  | RNA, testes | *0.0091*/***0.0014*** |
| C1046A | 39/11^1^; 10/6^2^ | Lifespan | 0.6801 |
|  |  | RNA, embryos | ***0.0018***/*0.0195* |
|  |  | RNA, heads | *0.0356*/***0.0091*** |
|  |  | RNA, testes | *0.0091*/***0.0014*** |
| **A1050G** | 26/24^1^; 11/5^2^ | Lifespan | *0.0226* |
|  |  | RNA, embryos | 0.5153/0.8548 |
|  |  | RNA, heads | 0.9029/0.2550 |
|  |  | RNA, testes | 0.9190/0.1039 |
| **C1177T** | 47/3^1^; 14/2^2^ | Lifespan | *0.0084* |
|  |  | RNA, embryos | ***0.0033***/***0.0037*** |
|  |  | RNA, heads | ***0.0021***/***0.0021*** |
|  |  | RNA, testes | *0.0121*/*0.0044* |
| -1180CA | 42/8^3^ | Lifespan | 0.2072 |
|  |  | RNA, embryos | - |
|  |  | RNA, heads | - |
|  |  | RNA, testes | - |
| C1183T | 45/5^3^ | Lifespan | 0.6331 |
|  |  | RNA, embryos | - |
|  |  | RNA, heads | - |
|  |  | RNA, testes | - |
| A1320- | 38/12^1^; 13/3^2^ | Lifespan | 0.1878 |
|  |  | RNA, embryos | 0.2992/0.2005 |
|  |  | RNA, heads | 0.2183/0.227 |
|  |  | RNA, testes | 0.4115/0.2333 |
| **G1991A** | 46/4^1^; 13/3^2^ | Lifespan | ***0.0028*** |
|  |  | RNA, embryos | 0.2992/0.2005 |
|  |  | RNA, heads | 0.2183/0.2274 |
|  |  | RNA, testes | 0.4115/0.2333 |
| **T1658C** | 45/5^1^; 13/3^2^ | Lifespan | *0.0195* |
|  |  | RNA, embryos | 0.1842/0.2881 |
|  |  | RNA, heads | 0.1111/0.1475 |
|  |  | RNA, testes | 0.1407/0.0535 |
| G2006A | 45/5^3^ | Lifespan | 0.5459 |
|  |  | RNA, embryos | - |
|  |  | RNA, heads | - |
|  |  | RNA, testes | - |
| G2038A | 35/15^1^; 11/5^2^ | Lifespan | 0.4075 |
|  |  | RNA, embryos | 0.8548/0.4161 |
|  |  | RNA, heads | 0.3932/0.6547 |
|  |  | RNA, testes | 0.6992/0.3711 |
| T2044G | 30/20^1^; 10/6^2^ | Lifespan | 0.1430 |
|  |  | RNA, embryos | 0.4957/0.0941 |
|  |  | RNA, heads | 0.3305/0.7853 |
|  |  | RNA, testes | 0.9689/0.7555 |
| C2058G | 34/16^1^; 11/5^2^ | Lifespan | 0.8853 |
|  |  | RNA, embryos | 0.5023/0.6112 |
|  |  | RNA, heads | 0.0736/0.0615 |
|  |  | RNA, testes | 0.1222/0.9029 |
| **433 + 871 + 1050 + 1177** |  | Lifespan | ***0.0042*** |
|  |  | RNA, embryos | - |
|  |  | RNA, heads | - |
|  |  | RNA, testes | - |
| **871 + 1050 + 1177** |  | Lifespan | ***0.0021*** |
|  |  | RNA, embryos | - |
|  |  | RNA, heads | - |
|  |  | RNA, testes | - |
| **871 + 1177, CC/GC/GT** | 4/43/3^1^; 3/11/2^2^ | Lifespan | ***0.0010*** |
|  |  | RNA, embryos | ***0.0001***/***0.0014*** |
|  |  | RNA, heads | ***0.0011***/***0.0016*** |
|  |  | RNA, testes | *0.0053*/*0.0125* |
| 871C+ 926G+ (1021 + 1046) + 1177 |  | Lifespan | - |
|  |  | RNA, embryos | ***0.0005***/***0.0042*** |
|  |  | RNA, heads | ***0.0058***/***0.0054*** |
|  |  | RNA, testes | *0.0053*/*0.0125* |

^1^ Data for the sample of 50 lines.

^2^ Data for the sample of 16 lines.

^3^ This marker does not segregate in a sample of 16 lines.

^4^ For associations with lifespan, P values of Wilkoxon test of line means, and for associations with *Lim3* transcription, P values of Wilkoxon test of mRNA amounts/C(t) are shown, see text for details.

Significant P values are in italics; P values surviving FDR correction are in italics and bold case; P values surviving Bonferroni correction are in italics, bold case and underlined.

Markers and haplotypes significantly associated with lifespan are in bold case.
